# Deciphering the Role of LNX2 as a Potential Contributor to Neurodevelopmental Disorders

**DOI:** 10.3390/genes17080849

**Published:** 2026-07-23

**Authors:** Mirella Vinci, Maria Grazia Figura, Antonino Musumeci, Miriam Virgillito, Valentina Finocchiaro, Simone Treccarichi, Alda Ragalmuto, Antonio Fallea, Concetta Federico, Salvatore Saccone, Francesco Calì

**Affiliations:** 1 Oasi Research Institute-IRCCS, 94018 Troina, Italy; mvinci@oasi.en.it (M.V.); mfigura@oasi.en.it (M.G.F.); amusumeci@oasi.en.it (A.M.); mvirgillito@oasi.en.it (M.V.); vfinocchiaro@oasi.en.it (V.F.); aragalmuto@oasi.en.it (A.R.); afallea@oasi.en.it (A.F.); cali@oasi.en.it (F.C.); 2 Department of Biological, Geological and Environmental Sciences, University of Catania, 95124 Catania, Italy; concetta.federico@unict.it (C.F.); salvatore.saccone@unict.it (S.S.); 3 Department Medicine and Surgery, Kore University of Enna, 94100 Enna, Italy

**Keywords:** E3 ubiquitin protein ligase, attention-deficit/hyperactivity disorder, neurodevelopmental disorders, next-generation sequencing, ligand of numb protein-X

## Abstract

Background/Objectives: Attention-deficit/hyperactivity disorder (ADHD) is a common neurodevelopmental condition characterized by a complex and multifactorial genetic architecture. In this study, we report a male patient, born to non-consanguineous healthy parents, presenting with ADHD and oppositional defiant disorder (ODD). Methods: Trio-based whole-exome sequencing (WES) was performed in the proband and both parents. Variant classification was performed according to American College of Medical Genetics and Genomics (ACMG) guidelines, and the potential pathogenicity of the identified variant was further assessed through multiple in silico prediction algorithms and protein structural analyses. Results: WES identified a homozygous variant in the *LNX2* gene (NM_153371.4: c.1165G>A, p.Ala389Thr), classified as a variant of uncertain significance (VUS) and supported by multiple in silico predictions. *LNX2* is expressed during brain development and encodes an E3 ubiquitin ligase involved in neuronal differentiation and synaptic function. The identified variant is located within the PDZ2 domain, a functionally relevant region involved in protein–protein interactions. Although the variant is reported in population databases (gnomAD ID: rs148429804), it has not been associated with any clinical phenotype, and its presence in the homozygous state has been reported only once, remaining extremely rare and lacking clinical annotation. Structural modelling predicted localized rearrangement of the hydrogen-bonding network within the PDZ2 domain without major conformational changes. Integrative transcriptomic, and single-cell analyses further supported the biological relevance of *LNX2* in neurodevelopment, highlighting its preferential association with neuronal projection-cell networks, synaptic vesicle trafficking pathways, and neuron-specific regulatory programs. Conclusion: Although the identified LNX2 variant cannot be considered causative for the patient’s phenotype and a definitive disease–gene relationship cannot be established based on a single individual, the complementary genetic, structural, and transcriptomic findings support the biological plausibility of LNX2 as a candidate gene for neurodevelopmental disorders. Additional independent patients and functional studies will be required to clarify its contribution to human disease.

## 1. Introduction

ADHD is a common neurodevelopmental condition characterized by a complex genetic architecture [1,2]. It is typically polygenic and frequently comorbid with other psychiatric disorders, including anxiety, depression, obsessive–compulsive disorder (OCD), and oppositional defiant disorder (ODD) [3,4]. Evidence suggests that dysregulation of the ubiquitin–proteasome system (UPS), particularly involving E3 ubiquitin ligases, may contribute to the pathophysiology of ADHD [5]. E3 ligases play a critical role in neuronal development and synaptic plasticity by regulating the stability and turnover of key proteins involved in neurotransmission, including those within dopaminergic and glutamatergic pathways. Alterations in ubiquitination processes can disrupt synaptic homeostasis, neuronal connectivity, and signaling pathways that are essential for attention, impulse control, and executive function [6,7]. Consistently, several genes encoding E3 ligases or related components have been implicated in neurodevelopmental disorders, supporting a potential link between ubiquitination defects and ADHD-related phenotypes [8,9,10]. This complexity reflects a broader paradigm shared across neurodevelopmental disorders, where heterogeneous genetic architectures—including rare variants, copy number variations, and polygenic contributions—interact with environmental and epigenetic factors to shape the phenotype [11].

**Figure 1:**
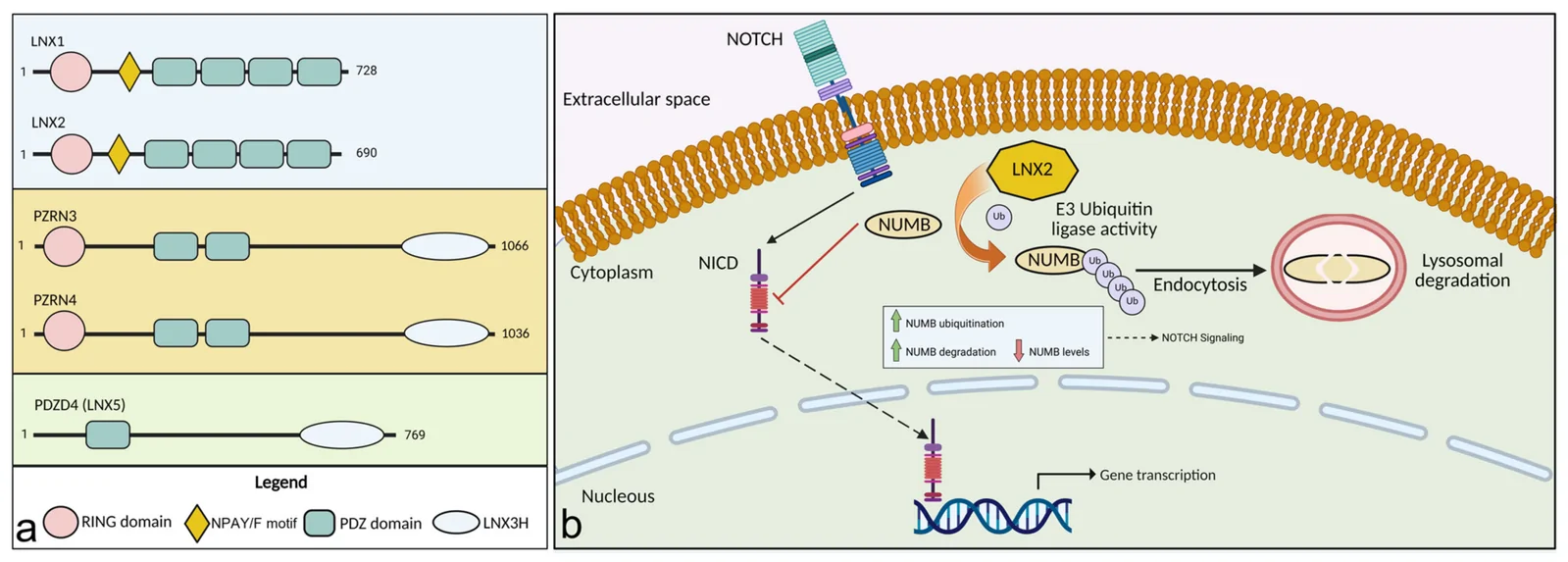
Domain organization of the LNX protein family and mechanistic model of LNX2-mediated regulation of NOTCH signaling through NUMB ubiquitination. (**a**) Schematic representation of the domain architecture of LNX family members, including LNX1, LNX2, PZRN3 (LNX3), PZRN4 (LNX4), and PDZD4 (LNX5). LNX1 and LNX2 display a conserved organization characterized by an N-terminal RING domain, followed by an NPXY motif and multiple PDZ domains, consistent with their role as E3 ubiquitin ligases mediating protein–protein interactions. In contrast, PZRN3 and PZRN4 contain fewer PDZ domains and an additional C-terminal LNX3 homology (LNX3H) domain, indicating structural diversification within the family. PDZD4 (LNX5) lacks the RING domain, suggesting the absence of E3 ubiquitin ligase activity. Protein lengths (amino acid residues) are indicated for each member. Domain types are represented as follows: RING domain (circle), NPAY/F motif (diamond), PDZ domains (rectangles), and LNX3H domain (oval). Figure 1a was adapted from a previous works [12,13]. (**b**) Mechanistic model of LNX2-mediated regulation of NOTCH signaling through NUMB ubiquitination. LNX2, an E3 ubiquitin ligase, promotes the ubiquitination of NUMB, leading to its endocytosis and lysosomal degradation. As NUMB functions as a negative regulator of NOTCH signaling, its depletion results in reduced inhibition of the NOTCH receptor. This facilitates the release of the NOTCH intracellular domain (NICD), which translocates to the nucleus and activates gene transcription. The overall effect is an enhancement of NOTCH signaling, potentially impacting downstream neurodevelopmental processes. Figure 1b was adapted from a previous works [13,14].

Among genes encoding E3 ubiquitin ligases, the Ligand of Numb protein-X (LNX) family, also known as the PDZRN family, comprises RING-type E3 ubiquitin ligases (*LNX1*–*LNX4*), whereas *LNX5* (also named PDZD4) lacks the RING domain and may not possess ubiquitin ligase activity. Figure 1 schematically illustrates the domain organization of the LNX protein family.

This gene family originated early during metazoan evolution and has been highly conserved across vertebrates, suggesting the acquisition of specialized functions [12,13]. From an evolutionary perspective, LNX genes belong to a conserved family of PDZ domain-containing E3 ubiquitin ligases that underwent diversification through gene duplication and domain rearrangement events, leading to the functional specialization observed in vertebrates [15]. Evolutionary and comparative analyses indicate that LNX proteins combine catalytic ubiquitination activity, mediated by the RING domain, with extensive protein–protein interactions driven by PDZ domains, which have undergone expansion and functional rewiring during vertebrate evolution [16,17]. This structural organization supports a conserved role for LNX proteins in regulating protein turnover and signaling pathways, particularly within the nervous system.

Across LNX genes, *LNX2* also known as PDZRN1, is widely expressed at mRNA level across adult tissues, including the central nervous system. Recent evidence has identified *LNX2* as a critical regulator of neurogenesis and neuronal differentiation [18]. Structurally, LNX2 shares a conserved architecture with LNX1 and comprises multiple functional domains, including a RING-type zinc finger domain, four PDZ domains, an NPAY/F motif, and intrinsically disordered regions. As an E3 ubiquitin ligase, LNX2 mediates the ubiquitination of target proteins, thereby regulating their stability and functional activity [16]. The RING domain is responsible for catalytic ubiquitin transfer, while the PDZ domains confer substrate specificity through protein–protein interactions. Notably, the presence of four PDZ domains enables LNX2 to interact with a broad range of binding partners, including Notch-associated components, supporting its involvement in key neuronal processes such as synapse formation, neurotransmission, and neuroglial regulation [12,13]. LNX2 is known to interact with the NUMB protein, via an NPAF motif, as well as with its paralog NUMBL (Numb-like). NUMB is a well-characterized cell fate determinant that regulates the Notch signaling pathway, which plays a critical role in vertebrate neurogenesis. Despite these established molecular interactions, the precise in vivo function of LNX2 during development remains to be fully elucidated [12,13]. LNX2 promotes substrate ubiquitination through the canonical E1–E2–E3 cascade. Upon substrate binding, LNX2 facilitates the transfer of ubiquitin from the E2 enzyme to lysine residues on the target protein [13]. The RING domain mediates the recruitment of ubiquitin-conjugating enzymes (E2) by functioning as a scaffold that facilitates the direct transfer of ubiquitin from the E2 enzyme to substrate proteins. The PDZ domains confer substrate specificity by recognizing C-terminal PDZ-binding motifs, thereby enabling LNX2 to interact with a broad spectrum of target proteins [13].

LNX2 also regulates the turnover and trafficking of membrane-associated proteins, particularly those containing PDZ-binding motifs. Ubiquitination of such proteins can promote their internalization and subsequent lysosomal degradation, contributing to the dynamic remodeling of cell–cell junctions and membrane signaling complexes [16].

The wide range of molecular interactions attributed to *LNX2* suggests that its functional role in the nervous system extends beyond cell fate determination. Indeed, *LNX2* may also be involved in synapse formation, regulation of neurotransmission, and modulation of neuroglial interactions [16]. Experimental studies have shown that *LNX2* knockout results in reduced anxiety-like behavior and increased risk-taking in mice, suggesting a role for *LNX2* in the regulation of neural circuits underlying behavioral control and emotional processing [19].

The present study aimed to explore the potential involvement of LNX2 in neurodevelopment by investigating the possible relationship between the clinical phenotype of a patient with ADHD/ODD and a rare homozygous LNX2 variant. By integrating genetic findings with structural modelling and publicly available transcriptomic datasets, we sought to assess the biological plausibility of LNX2 as a candidate gene for neurodevelopmental disorders. Although the available evidence does not establish a causal disease–gene relationship, our findings support the hypothesis that the ubiquitin E3 ligase pathway, and LNX2 in particular, may contribute to neurodevelopmental processes relevant to these conditions.

## 2. Materials and Methods

### 2.1. WES Analysis

Genomic DNA was obtained from peripheral blood leukocytes of the proband and both parents, using a well-established protocol [20]. Libraries were prepared for the Trio-based WES analysis through the Agilent SureSelect V7 Kit (Santa Clara, CA, USA), according to the manufacturer’s instructions. Sequencing was performed on the Illumina HiSeq 3000 (San Diego, CA, USA), achieving a minimum coverage of 20× for 97% of the targeted regions. Variant filtering was conducted based on (i) presumed inheritance patterns—recessive, de novo, or X-linked—and (ii) a minor allele frequency (MAF) below 1%, referencing population databases including 1000 Genomes, ESP6500, ExAC, and gnomAD. This threshold was applied as an initial screening criterion to account for population-specific genetic variability. Subsequently, variants absent or extremely rare in population databases were prioritized for further analysis. The overall analytical workflow is consistent with current next-generation sequencing strategies applied in clinical genomic diagnostics, particularly in trio-based WES approaches, including variant prioritization based on inheritance models and population frequency [21]. The human reference genome assembly GRCh38 (hg38) was used for sequence alignment and variant analysis. Candidate variants were subsequently prioritized according to presumed mode of inheritance (recessive, de novo, or X-linked), predicted functional impact, and consistency with the patient’s clinical phenotype as defined by Human Phenotype Ontology (HPO) terms, including elevated frustration (HP:0033676), attention-deficit/hyperactivity disorder (ADHD; HP:0007018), low frustration tolerance (HP:0033677), hyperactive behavior (HP:0000752), oppositional defiant behavior/disorder (HP:0033057), and autism spectrum disorder (ASD; HP:0000729). All the prioritized variants were confirmed by Sanger sequencing. The reaction was performed with the BigDye™ Terminator v1.1 Cycle Sequencing Kit (Life Technologies, Carlsbad, CA, USA) on the SeqStudio Genetic Analyzer (Thermo Fisher Scientific, Waltham, MA, USA).

### 2.2. Data Analysis

Common variants and non-exonic polymorphisms were excluded by applying a minor allele frequency (MAF) threshold of <1% using public databases, including gnomAD v4.1.0, the 1000 Genomes Project, and the Exome Sequencing Project (accessed on 16 March 2026). Variants previously reported as pathogenic or likely pathogenic were investigated using the Human Gene Mutation Database (HGMD Professional 2026.1) (accessed on 16 March 2026). Variant filtering, annotation, and prioritization were performed using Franklin by QIAGEN (Hilden, Germany) on the generated VCF files. The variant unveiled by WES was classified using the “American College of Medical Genetics” (ACMG) guidelines and criteria [22]. The ClinVar database (https://www.ncbi.nlm.nih.gov/clinvar) (accessed on 9 March 2026) was queried to identify genetic variants in *LNX2* gene and was subsequently used for the submission of the identified variant. DOMINO [23] (https://domino.iob.ch/) (accessed on 9 March 2026) was used for predicting the inheritance pattern of *LNX2* gene. DOMINO generates a score ranging from 0 to 1, with lower scores indicating a higher likelihood of autosomal recessive inheritance and higher scores indicating a higher likelihood of autosomal dominant inheritance. Pathogenicity prediction was performed using multiple in silico algorithms available through the VarSome platform [24] (accessed on 16 March 2026). Since MutationTaster was not included in the VarSome output, its pathogenicity prediction was performed independently using the MutationTaster web server (https://www.mutationtaster.org/) (accessed on 9 March 2026), and the resulting score was reported separately.

The AlphaFold3 server (https://alphafoldserver.com/) (accessed on 16 March 2026) was used to predict the structural effects of the *LNX2* variant identified by WES. For each protein, the best-ranked model was selected based on the highest predicted Local Distance Difference Test (pLDDT) score. The AlphaFold3 structural models generated in this study are available as Supplementary Files (File S1 and S2). To ensure reproducibility of the structural analyses, the experimentally determined reference structure of the LNX2 PDZ2 domain (PDB ID: 5E11; DOI: 10.2210/pdb5e11/pdb) has also been included as Supplementary File (File S3). Protein structural analyses and visualization were performed using UCSF ChimeraX version 1.10.1. Protein stability changes associated with the p.Ala389Thr variant were predicted using the computational algorithm mCSM (https://biosig.lab.uq.edu.au/mcsm/stability) (accessed on 16 March 2026) and MuPro (https://mupro.proteomics.ics.uci.edu/) (accessed on 16 March 2026). MUpro predicts whether an amino acid substitution increases or decreases protein stability. Negative confidence scores indicate decreased protein stability, whereas positive scores indicate increased stability, with values farther from zero reflecting greater prediction confidence. The effect of the variant on protein flexibility and intrinsic disorder was assessed using IUPred2 (https://iupred2a.elte.hu/) (accessed on 16 March 2026). IUPred2 assigns a score ranging from 0 to 1 to each amino acid residue, with lower values indicating ordered regions and higher values indicating intrinsically disordered regions. Multiple sequence alignment of human LNX2 (UniProt ID: Q8N448) and LNX1 (UniProt ID: Q8TBB1) proteins was performed using ClustalW implemented in BioEdit software (version 7.7). The Hardy–Weinberg equilibrium principle was applied to estimate the expected frequency of the homozygous variant in the gnomAD global population. Expected genotype frequencies were calculated under Hardy–Weinberg equilibrium using the equation:

p^2^ + 2pq + q^2^ = 1

where (q) represents the frequency of the mutant LNX2 allele (0.0003104) and (p = 1 − q).

Subsequently, a theoretical Bayesian estimate of the probability of observing the homozygous genotype among individuals with ADHD was calculated using the formula:

P(Hom∣ADHD) = [P(ADHD∣Hom)∙P(Hom)]/P(ADHD).

### 2.3. Integrative Transcriptomic, Co-Expression, and Epigenomic Analyses

To further investigate the potential neurobiological relevance of LNX2 in neurodevelopmental disorders, publicly available transcriptomic and epigenomic datasets retrieved from Gene Expression Omnibus (GEO) (https://www.ncbi.nlm.nih.gov/geo/) (accessed on 8 May 2026) and PsychENCODE/PsychSCREEN (https://psychscreen.beta.wenglab.org/psychscreen) (accessed on 8 May 2026) were interrogated. Differential expression and co-expression analyses were performed using ASD postmortem brain transcriptomic datasets GSE28521 and GSE38322, selected for their region-specific brain profiles and adequate biological replication. To ensure reproducibility, the complete differential expression output and the corresponding co-expression gene lists for the GSE28521 and GSE38322 datasets are provided as Supplementary Material (Tables S1 and S2, respectively). An exploratory evaluation of the ADHD-related dataset GSE52889 was also conducted, although results were interpreted descriptively due to pooled lymphoblastoid samples and limited biological replication. Functional enrichment analyses of LNX2-associated co-expression modules were performed using Gene Ontology (GO) biological process annotations. Single-cell transcriptomic analyses were conducted using PsychENCODE/PsychSCREEN snRNA-seq datasets derived from dorsolateral prefrontal cortex (DLPFC) samples to evaluate cell type-specific LNX2 expression across neuronal and glial populations. Bubble plot visualizations were generated in R (version 4.5.2) using the ggplot2 package.

## 3. Results

### 3.1. Case Presentation

The patient is an only male child of non-consanguineous, healthy parents. The pregnancy was uneventful; however, delivery was complicated by an urgent dystocic birth due to the presence of two nuchal cord loops and oligohydramnios. Birth weight was 2.5 kg, and length was 49 cm.

In the neonatal period, the patient was admitted to the Neonatology Unit at 24 h of life for a 2-day hospitalization for rehydration. Physiological jaundice was reported, with no further complications described. He began babbling at 6 months and produced his first words before the age of one year; subsequent language development was normal.

Developmental milestones were overall within normal limits. Motor development was reported as regular, with independent walking achieved at 12 months of age. Bladder and bowel control were acquired at approximately 3 years of age. He did not exhibit sleep disturbances during the first years of life. According to parental reports, early behavioral features were already evident from approximately 17 months of age, when the patient showed reduced interest in interacting with peers and a preference for older children. During preschool, he progressively developed socialization difficulties, oppositional behaviors, poor frustration tolerance, emotional dysregulation, and increasing disruptive behaviors in the classroom, eventually leading to the clinical diagnosis of ADHD and oppositive defiant disorder (ODD).

From a behavioral and neuropsychiatric standpoint, the patient exhibited difficulties in social interactions, characterized by oppositional behaviors toward both parents and teachers, low frustration tolerance, and a tendency toward defiant attitudes. Episodes of aggressive behavior directed mainly toward the parents were also reported. The patient shows difficulty accepting defeat and may display domineering behaviors in peer interactions. Furthermore, emotional dysregulation is evident, particularly in managing positive emotions. Neurological examination was normal. No significant dysmorphic features were observed. Oral and dental assessment revealed no clinically significant abnormalities. The EEG did not show epileptiform abnormalities.

Based on the clinical presentation, a suspected diagnosis of ADHD and oppositional defiant disorder (ODD) was considered. Although there were features suggestive of a diagnosis of autism spectrum disorder (ASD) (including social atypicalities and repetitive behaviors), the criteria for a formal diagnosis were not fully met. Array comparative genomic hybridization (array-CGH) analysis performed in the patient revealed no clinically significant copy number variants.

### 3.2. WES Analysis

**Figure 2:**
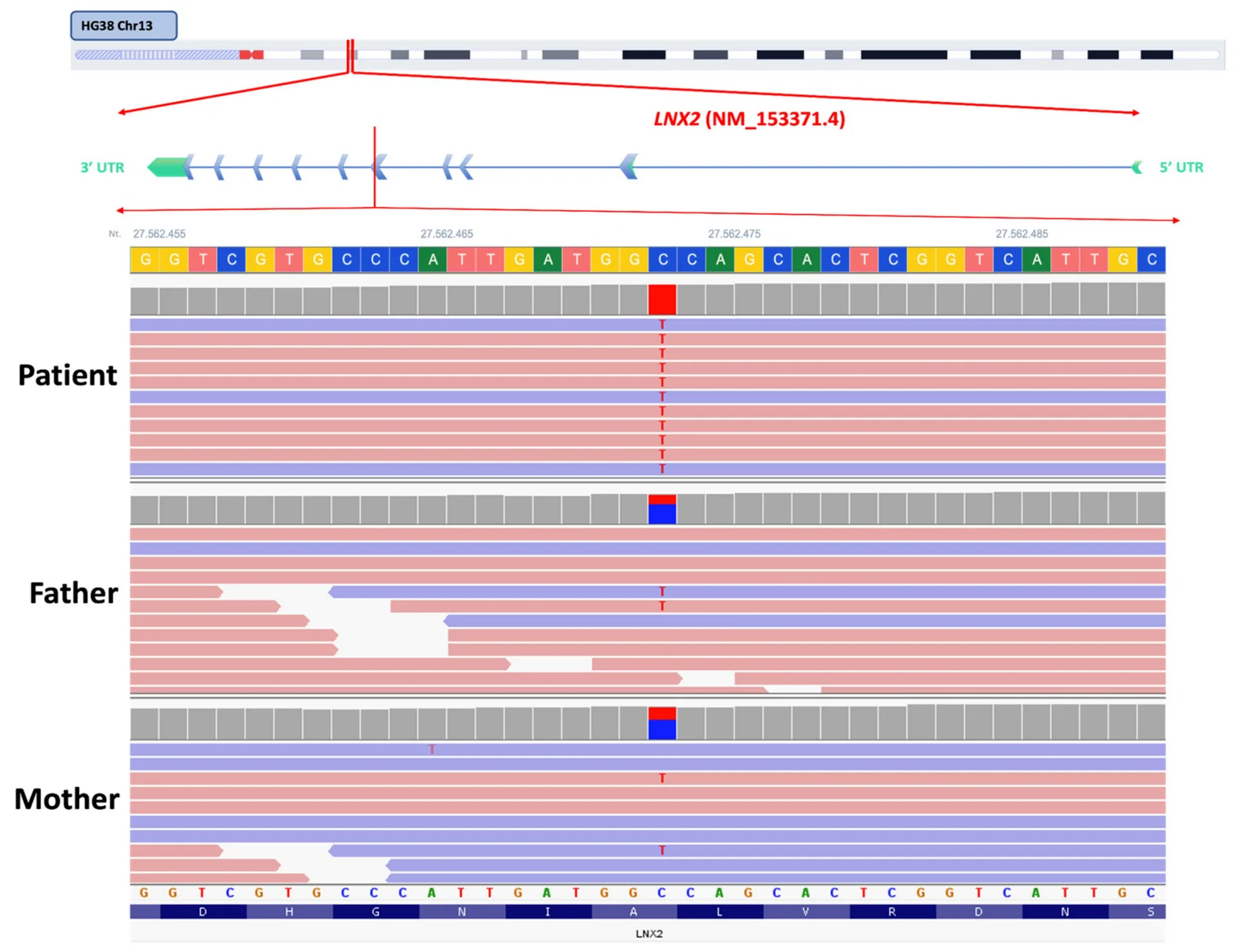
Genomic localization of the *LNX2* gene and the c.1165G>A variant identified in this study. The figure also includes Integrative Genomics Viewer (IGV) visualization of the inherited homozygous c.1165G>A variant in *LNX2* (NM_153371.4).

WES identified a homozygous nucleotide substitution, c.1165G>A, in exon 5 of the LNX2 gene (NM_153371.4). Figure 2 shows the chromosomic localization of the LNX2 gene as well as its Integrative Genomics Viewer (IGV) visualization of the identified genetic variant.

Additionally, a pathogenic variant in *HBB HBB* (NM_000518.5) (c.92+6T>C; dbSNP ID: rs35724775), previously associated with β-thalassemia, was detected and found to be inherited from the father. Given its established association with a distinct clinical condition, the *HBB* variant is unlikely to explain the patient’s neurological phenotype. Therefore, the *LNX2* variant is considered a potential candidate for the patient’s clinical presentation. No additional rare biallelic, de novo, or clinically relevant pathogenic variants affecting established neurodevelopmental genes were identified through trio-based WES analysis. To strengthen the link between LNX2 and NDDs, especially ADHD, the identified variant was submitted to the ClinVar database (SCV007596023).

According to the DOMINO predictor, *LNX2* is predicted to have autosomal recessive inheritance transmission showing a score of 0.175 (score range from 0 for autosomal recessive to 1 for autosomal dominant). This homozygous variant has been classified as VUS according to the ACMG/AMP guidelines, based on the application of criteria PM2 (extremely low frequency in population data-bases) and BP4 (computational evidence supporting a benign effect on the gene or gene product). Several in silico prediction analyses support the classification of the variant as VUS (Table 1).

A detailed summary of the VarSome prediction results is provided in Supplementary Table S3. The nucleotide affected by the c.1165G>A variant is highly conserved across species, as indicated by the PhyloP100 score of 5.999, where higher positive values reflect stronger evolutionary constraint. This observation is further supported by a GERP++ RS score of 4.75, with positive scores indicating evolutionary conservation under purifying selection.

MutationTaster classified the LNX2:c.1165G>A variant as deleterious. The gnomAD database reports the presence of the variant in 501 alleles out of 1,614,170 total alleles. The mutated allele is equally distributed between male and females. Notably, only one homozygous individual has been reported, specifically within the European (non-Finnish) population. Based on the global allele frequency (q = 0.0003104), the expected frequency of homozygous individuals under Hardy–Weinberg equilibrium is q^2^ = 9.63 × 10^−8^, corresponding to approximately 1 homozygote per 10 million individuals. The variant is predominantly observed in the European (non-Finnish) population, where it shows a slightly higher allele frequency (q = 0.0003297). In this subgroup, the expected frequency of homozygous individuals is q^2^ = 1.09 × 10^−7^, corresponding to approximately 1 individual per 9.2 million.

### 3.3. In Silico Structural Analysis

The mutation p.Ala389Thr is located within the PDZ2 domain. The LNX2 protein is composed by 690 residues and contains a RING-type zinc finger domain (residues 50–88) (ProRule PRU00175), two disordered regions (residues 198–224 and 418–455), and an NPAY/F motif (residues 208–211). It also includes four PDZ domains (ProRule PRU00143) located at residues 233–318 (PDZ1), 339–422 (PDZ2), 468–554 (PDZ3), and 600–688 (PDZ4).

**Figure 3:**
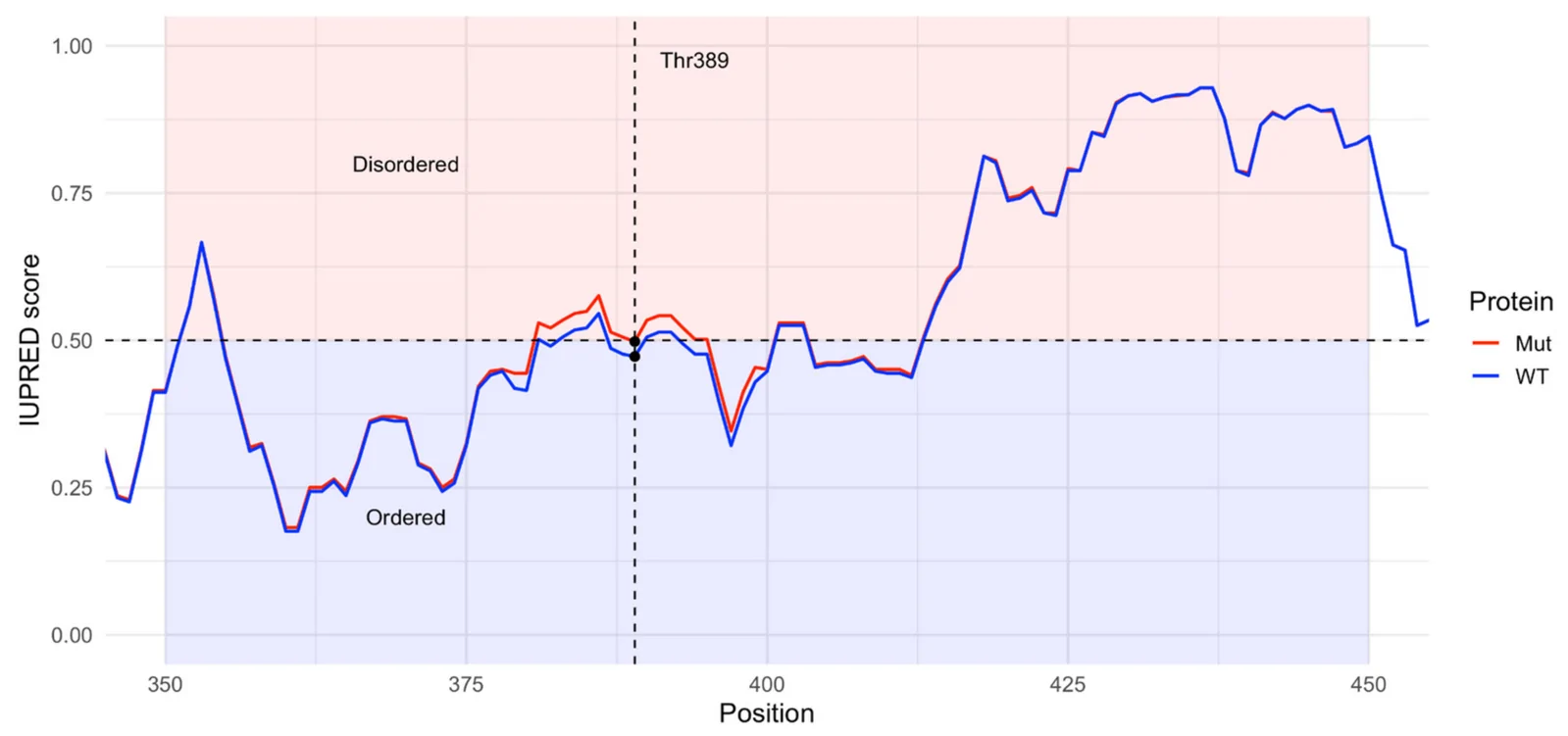
IUPred2 prediction of intrinsic disorder for the LNX2 wild-type and the p.Ala389Thr variant. IUPred2 prediction of intrinsic disorder for the wild-type LNX2 and the p.Ala389Thr variant. The disorder score per residue (0–1) is plotted along the protein region, with the disorder threshold set at 0.5. The dotted vertical line marks the position of the variant (Thr389), and the black dots indicate the position of the mutated amino acid. Both the wild-type and mutant profiles show similar trends, although an increase in the propensity for disorder is observed in the mutant around the site of the variant.

Moreover, MuPro analysis underlines that the variant decreases protein stability, as indicated by the ΔΔG score of −1.2798362 (score range: −1 to 1). Also mCSM algorithm predicted a destabilizing effect of the mutations with a reduction of the ΔΔG of −1.552 kcal/mol. The IUPred2A analysis shows an increase in intrinsic disorder in the region surrounding the mutation at amino acid position 389. Specifically, the amino acid residues at positions 382, 387, 388, 393, 394, and 395 show a shift from below to above the 0.5 threshold when comparing the wild-type to the mutated form (Figure 3).

**Figure 4:**
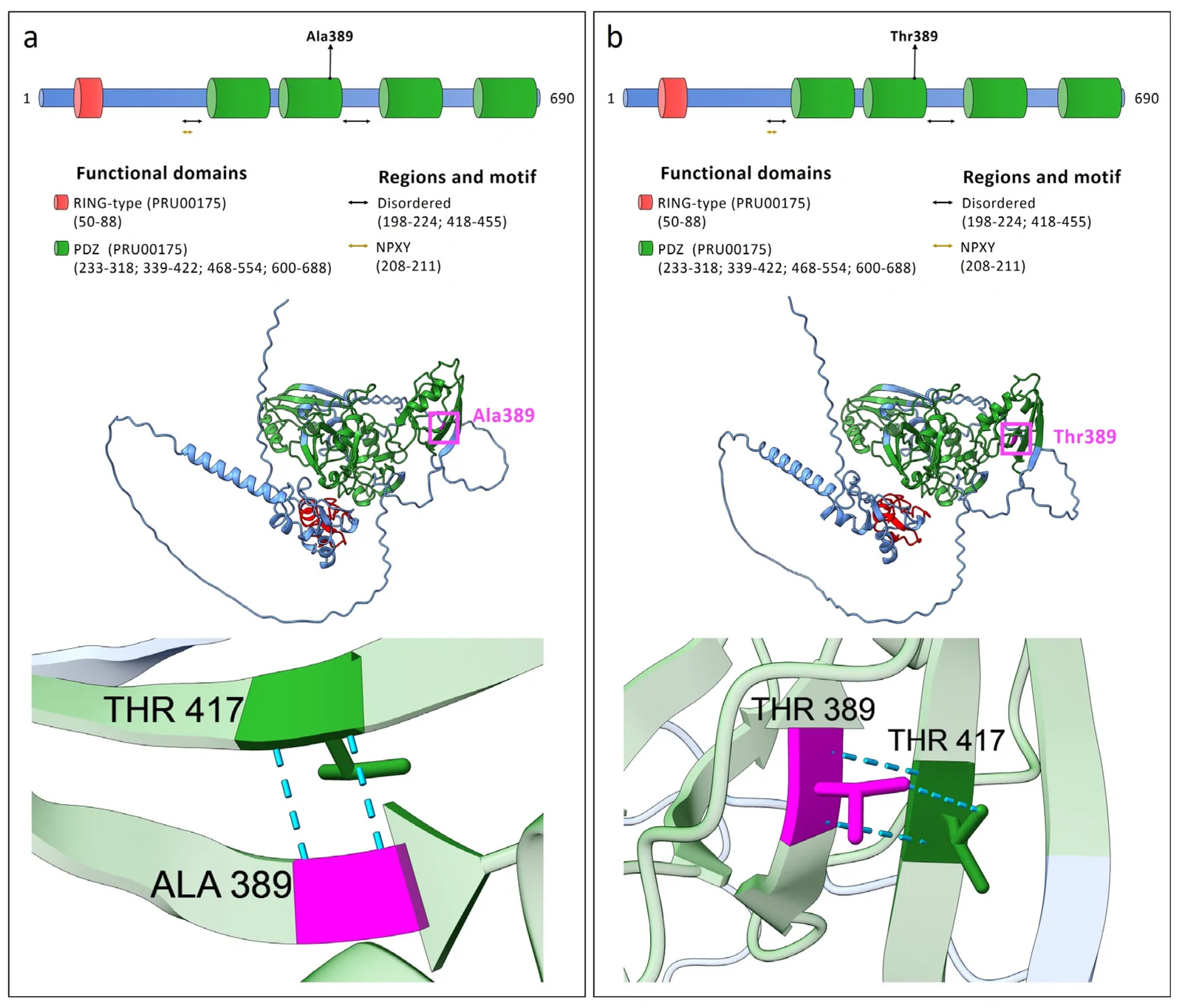
Structural impact of the p.Ala389Thr variant in LNX2. (**a**) Schematic representation of the wild-type LNX2 protein showing domain organization, including the RING-type domain, PDZ domains, NPXY motif, and intrinsically disordered regions. The corresponding AlphaFold3-predicted structure is shown below, with the position of Ala389 highlighted. A close-up view of the PDZ2 domain (residues 339–422) illustrates the local structural context, where Ala389 forms hydrogen bond interactions with Thr417. (**b**) Representation of the p.Ala389Thr mutant LNX2 protein. The substitution site is indicated within the PDZ2 domain in both the domain schematic and the predicted three-dimensional structure. A magnified view of the mutated region shows the introduction of Thr389 and its altered hydrogen bonding pattern with Thr417, resulting in a local rearrangement of the interaction network.

Structural modelling and structural superposition of the wild-type and p.Ala389Thr PDZ2 domains revealed no major conformational changes, with the overall domain architecture remaining highly conserved. However, replacement of alanine with threonine introduced a polar hydroxyl-containing side chain, enabling the formation of an additional local hydrogen bond with Thr417. Whereas the wild-type Ala389 established two backbone-mediated hydrogen bonds with Thr417, the mutant Thr389 was predicted to form an additional side-chain hydrogen bond involving its hydroxyl group (Figure 4).

Structural alignment revealed a high degree of similarity between the wild-type and p.Ala389Thr predicted models. Specifically, 389 residues were aligned with a root mean square deviation (RMSD) of 0.662 Å, indicating strong local structural similarity. However, when considering the entire protein (690 residues), the RMSD increased to 6.253 Å, suggesting broader conformational differences. Notably, the region encompassing the mutated residue did not align with the corresponding region in the wild-type structure (Figure S1).

### 3.4. Integrative Transcriptomic and Epigenomic Analyses of LNX2-Associated Neuronal Networks

Publicly available ASD transcriptomic datasets were interrogated to investigate the potential involvement of LNX2 in NDDs. In the postmortem brain dataset GSE28521, LNX2 displayed a modest trend toward reduced expression in frontal cortex samples from ASD subjects compared with controls, with a weaker reduction observed in temporal cortex tissue. Although these differences did not reach statistical significance, regional variation in LNX2 expression was observed across ASD cortical samples. Analysis of the independent ASD dataset GSE38322 revealed partially divergent regional trends, with modestly increased LNX2 expression detected in cerebellar and occipital cortex tissues from ASD subjects. Figure S2 shows the regional LNX2 expression across the independent ASD transcriptomic datasets GSE28521 and GSE38322.

**Figure 5:**
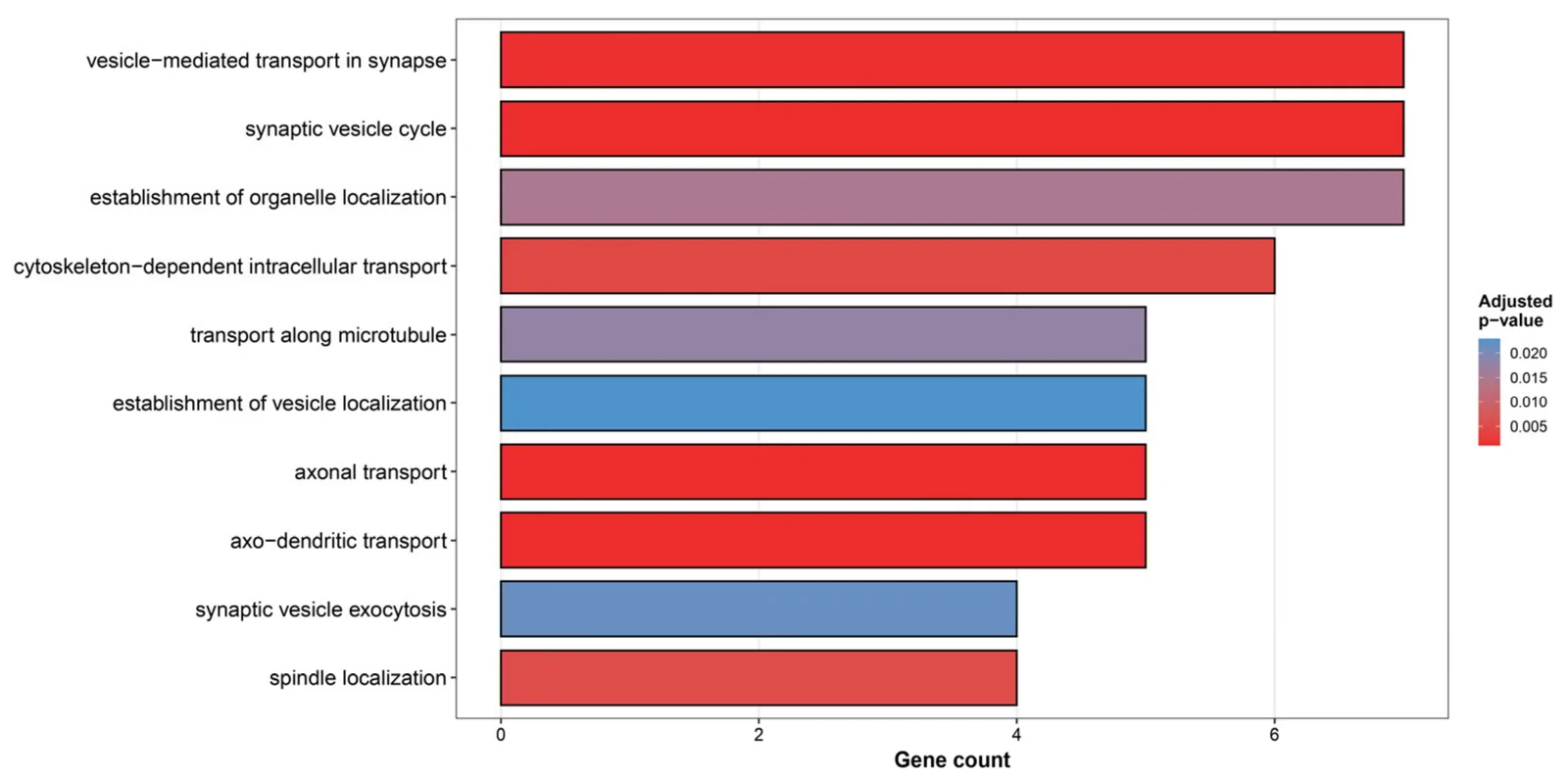
Functional enrichment of the LNX2 co-expression module in ASD frontal cortex. Gene Ontology enrichment analysis of genes positively correlated with LNX2 expression in GSE28521 revealed significant enrichment for biological processes related to synaptic vesicle cycling, synaptic vesicle transport, regulated exocytosis, axonal transport, and microtubule-dependent intracellular trafficking. Bar length indicates the number of genes associated with each biological process, while color intensity represents adjusted *p*-values.

To further characterize the biological context associated with LNX2 expression, co-expression analyses were performed across ASD brain datasets. In GSE28521, the LNX2-associated transcriptional module was enriched for genes involved in neuronal signaling, vesicle trafficking, intracellular transport, and cytoskeletal organization, including *PTPRT*, *KCND2*, *CADPS*, *SCAMP1*, *ATG5*, and *WASL*. Gene Ontology enrichment analyses identified pathways related to synaptic vesicle cycling, intracellular trafficking, axonal transport, and regulated exocytosis (Figure 5).

**Figure 6:**
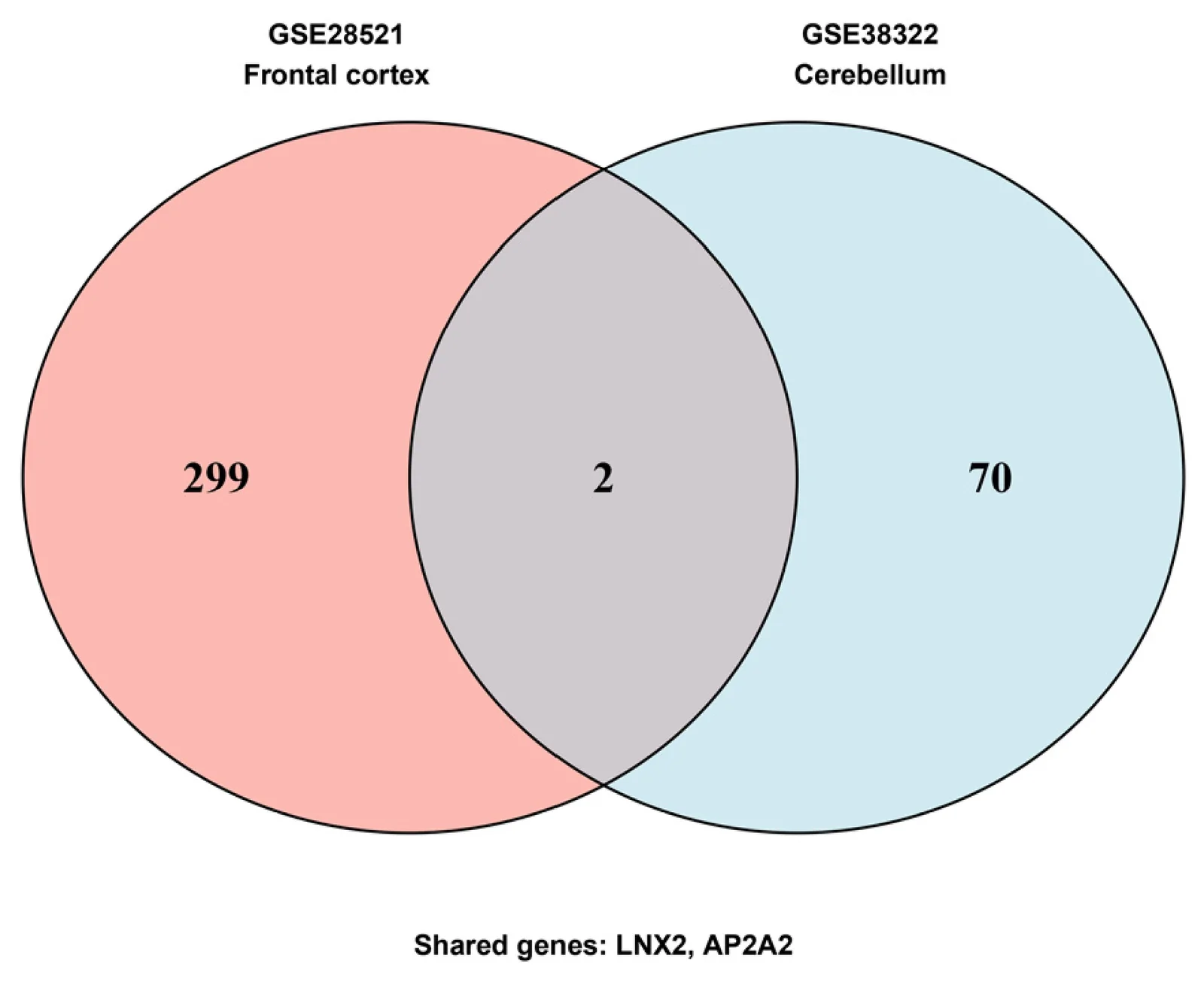
Overlap analysis of *LNX2*-associated co-expression modules across independent ASD transcriptomic datasets. Comparison between frontal cortex-associated (GSE28521) and cerebellar-associated (GSE38322) *LNX2* co-expression networks revealed limited overlap between highly correlated genes. Notably, AP2A2 emerged as a shared co-expressed gene across datasets. AP2A2 encodes a component of the adaptor protein-2 (AP-2) complex involved in clathrin-mediated endocytosis and synaptic vesicle recycling, further supporting the association of *LNX2* with neuronal trafficking and synaptic regulatory pathways.

In contrast, co-expression analysis of cerebellar samples from GSE38322 identified a more restricted enrichment profile. Gene Ontology analysis revealed significant enrichment for the biological process “neuronal stem cell population maintenance” (adjusted *p*-value = 0.0044), supported by five positively correlated genes within the LNX2-associated module: *HOOK3*, *SS18*, *MMP24*, *FOXO1*, and *JAG1*. Comparative network analysis demonstrated substantial regional heterogeneity between LNX2-associated transcriptional modules. Only two highly co-expressed genes overlapped between datasets: LNX2 itself and AP2A2 (Figure 6).

**Figure 7:**
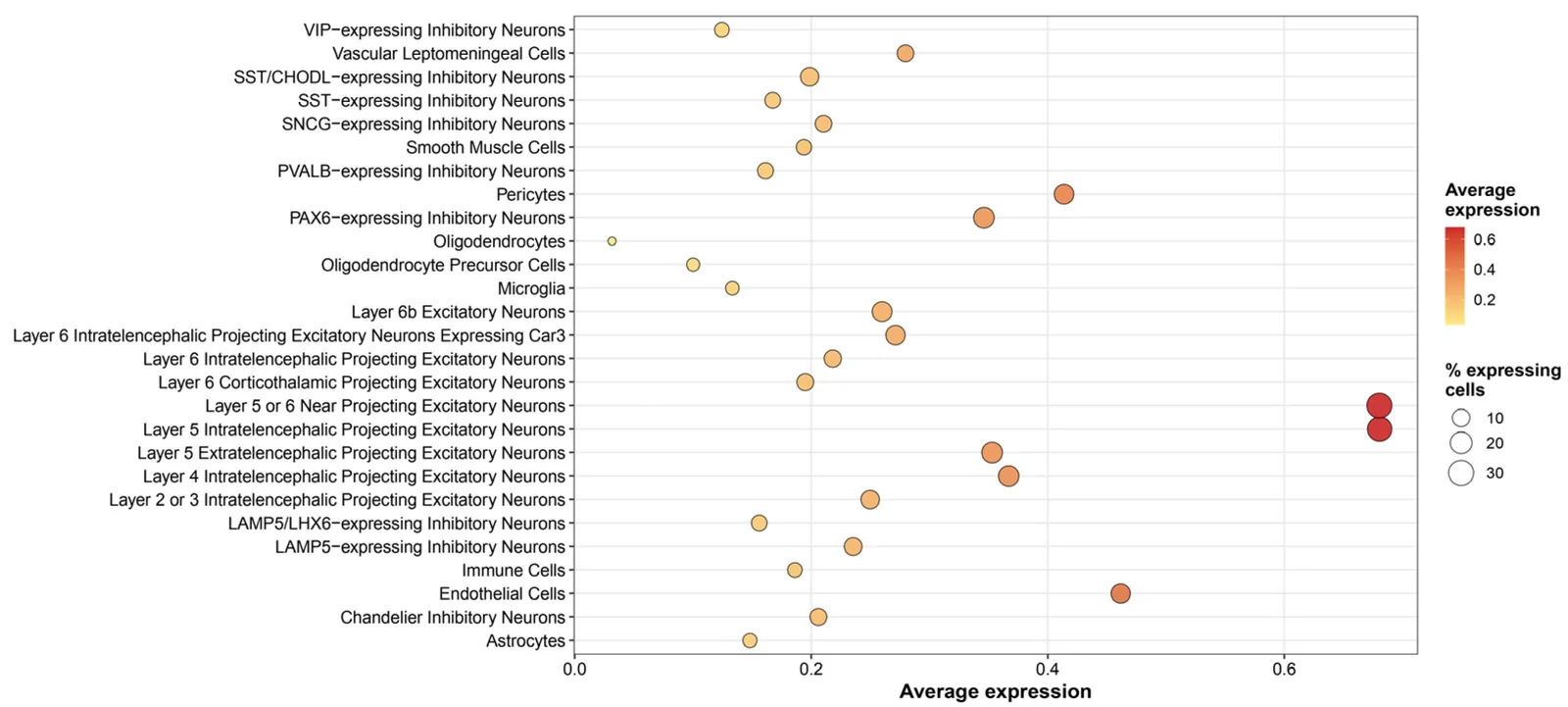
Cell type-specific expression profile of *LNX2* in a human dorsolateral prefrontal cortex (DLPFC) single-cell transcriptomic dataset derived from ASD (n = 27) and control (n = 25) adult DLPFC samples with integrated snRNA-seq and snATAC-seq data retrieved from PsychSCREEN. Bubble plot analysis demonstrates preferential LNX2 expression within deep-layer cortical excitatory projection neurons, particularly layer 5 intratelencephalic and layer 5/6 near-projecting excitatory neuronal populations. Additional expression was detected across selected inhibitory neuronal subtypes and vascular-associated cell populations. Dot size represents the percentage of cells expressing LNX2 within each cellular population, whereas color intensity indicates average normalized expression levels. The preferential enrichment of LNX2 within cortical projection neurons supports its potential involvement in neuronal connectivity, synaptic integration, and higher-order cortical circuitry relevant to NDDs.

Single-cell transcriptomic profiling of dorsolateral prefrontal cortex (DLPFC) samples derived from ASD and control subjects further demonstrated a non-uniform, cell type-specific expression pattern of LNX2. Preferential enrichment was observed within deep-layer cortical excitatory projection neurons, particularly layer 5 intratelencephalic and layer 5/6 near-projecting neuronal populations, with additional expression detected across selected inhibitory neuronal subtypes (Figure 7).

## 4. Discussion

In this work, we describe an individual presenting with ADHD and ODD. Trio-based WES prioritized a homozygous variant in the *LNX2* gene (NM_153371.4: c.1165G>A; p.A389T). Although the variant is currently classified as a VUS according to ACMG criteria, its localization within a functionally relevant PDZ domain, together with complementary structural, evolutionary, and transcriptomic analyses from public databases, supports the biological plausibility of LNX2 involvement in neurodevelopment. Nevertheless, these findings should not be interpreted as establishing a causal disease–gene relationship. The pathogenic variant identified in the *HBB* gene (NM_000518.5:c.92+6T>C) was communicated to the patient during genetic counseling; however, it was not considered causative of the clinical presentation. Given the complex and multisystemic nature of NDDs (especially ADHD) [25], we cannot exclude the possibility that additional genetic findings identified by WES, as well as non-coding intronic variants or epigenetic mechanisms, may contribute to the patient’s phenotype. This observation should be interpreted within the well-established multifactorial framework of neurodevelopmental disorders, in which rare variants may act as contributors within a broader polygenic and environmentally modulated background, rather than representing fully penetrant causal events [11]. A similar interpretative framework has been proposed in recent studies describing candidate genes in neurodevelopmental disorders, where single-case genomic findings, supported by in silico and structural analyses, contribute to expanding the spectrum of potentially relevant genes despite the absence of definitive genotype–phenotype correlations [26].

According to the DOMINO predictor, *LNX2* variants are more consistent with an autosomal recessive mode of inheritance. Although *LNX2* has been implicated in several processes related to neural development [16], no specific OMIM phenotype has yet been assigned to this gene. Gene constraint metrics retrieved from DECIPHER database, indicate that *LNX2* is relatively tolerant to loss-of-function variation, as reflected by a pLI score of 0.00, a loss-of-function observed/expected (LOEUF) value of 0.69, and a low sHet score. Consistently, the missense Z-score (1.18) suggests limited constraint against amino acid substitutions. These findings argue against a classical haploinsufficiency mechanism. In contrast, in silico protein-level predictions indicate a higher probability of dominant-negative (pDN = 0.627) or gain-of-function (pGOF = 0.685) effects compared to loss-of-function (pLOF = 0.379).

The variant identified in this study is classified as a VUS and is reported in the ClinVar database without an associated phenotype (ID: 2315524). Nevertheless, it should be noted that ClinVar does not report whether the variant has been observed in the homozygous state. The genetic variant that we found in the *LNX2* gene is also reported in both dbSNP (ID: rs148429804) and ClinGen (ID: CA6926922) databases. To strengthen the evidence linking LNX2 to neurodevelopmental disorders, particularly ADHD, the homozygous variant identified in our patient was deposited in the ClinVar database under submission number SCV007596023.

The identified *LNX2* variant is extremely rare in the general population. Based on the allele frequency reported in gnomAD for the European (non-Finnish) population (q ≈ 0.00031), the expected homozygous frequency under Hardy–Weinberg equilibrium is approximately 1 in 10 million individuals. As no phenotypic information is available for this individual, the clinical significance of this observation cannot be assessed. Nevertheless, the presence of an apparently healthy homozygous carrier argues against a fully penetrant Mendelian model and suggests that, if involved in disease, the identified variant may act as a susceptibility allele or exhibit in-complete penetrance and/or variable expressivity within the complex genetic architecture of NDDs.

In silico prediction algorithms classified the variant as VUS, although some algorithms, such as CADD and MutationTaster, suggested a pathogenic effect. Specifically, MutationTaster classified the LNX2 c.1165G>A (p.Ala389Thr) variant as “deleterious” in the MANE transcript, with a tree vote of 85/15 (deleterious | benign), indicating that 85% of the models support a deleterious effect. In addition, evolutionary conservation analyses provide further support for the functional relevance of the affected nucleotide. The high PhyloP100 score (5.999) and GERP score (4.75) indicate strong evolutionary constraint at this position, suggesting that substitutions at this site are likely to be deleterious.

Gene Ontology (GO) annotations indicate that the protein encoded by *LNX2* possesses ubiquitin-protein transferase activity (GO:0004842) and is involved in multiple protein interaction processes, including protein binding (GO:0005515), PDZ domain binding (GO:0030165), and identical protein binding (GO:0042802). Functionally, it is implicated in nervous system development (GO:0007399), while at the cellular level it is primarily localized to the plasma membrane (GO:0005886). These annotations suggest a role for LNX2 in ubiquitin-mediated protein regulation and signaling pathways relevant to neural development.

**Figure 8:**
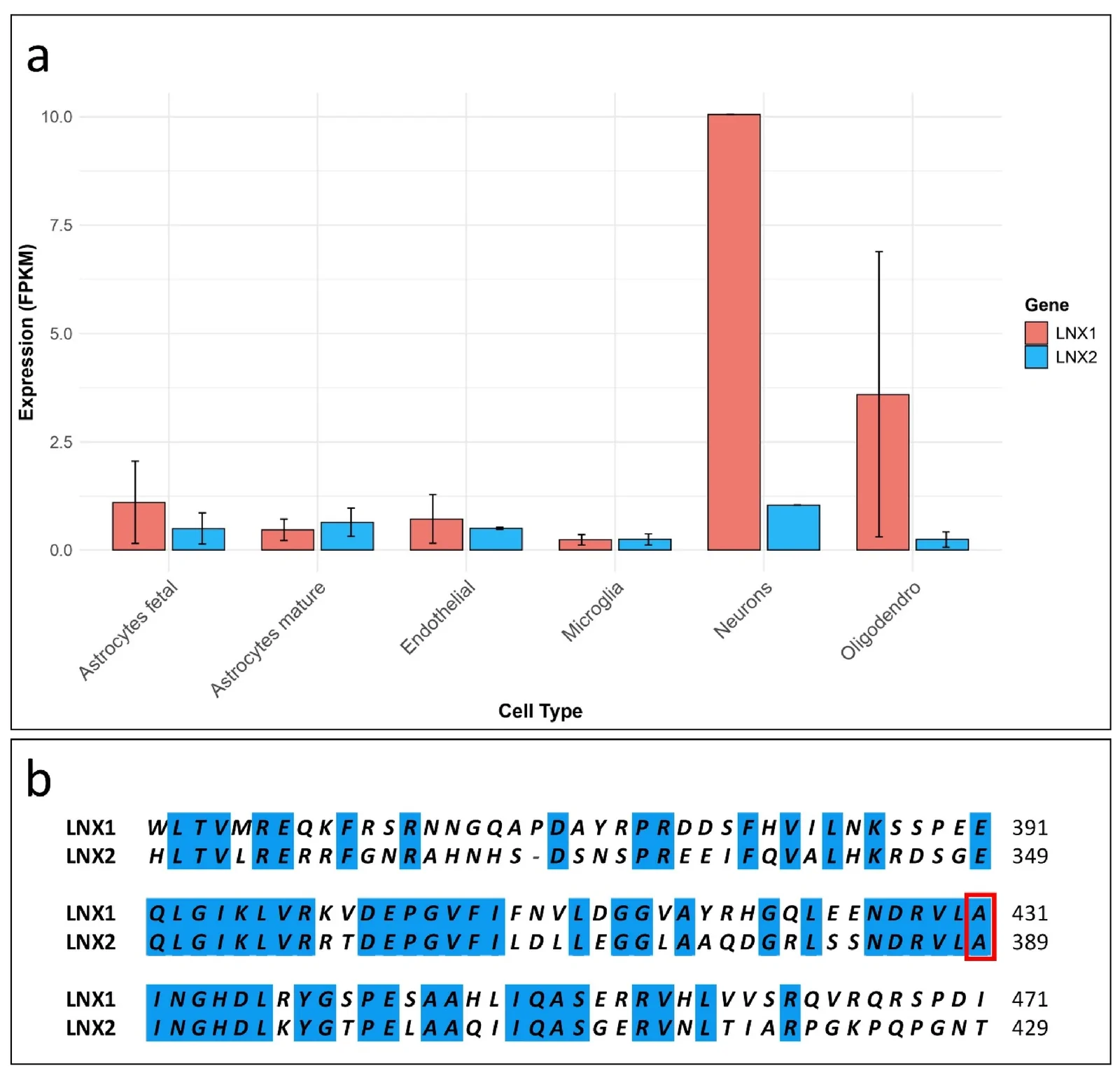
Expression profile and sequence conservation of *LNX1* and *LNX2*. (**a**) Gene expression levels of *LNX1* (red) and *LNX2* (blue) across different human brain cell types, including fetal astrocytes, mature astrocytes, endothelial cells, microglia, neurons, and oligodendrocytes. Expression is reported as FPKM values. Brain expression data has been retrieved from BrainRNAseq database. (**b**) Protein sequence alignment of *LNX1* and *LNX2* highlighting conserved regions within the PDZ2 domain. Conserved residues are indicated in blue, while the position corresponding to the variant identified in LNX2 (Ala389) is highlighted (red square).

A comparative analysis between *LNX1* and *LNX2* provides additional insights into the potential functional relevance of the identified variant. Although these genes are paralogues, they share a conserved molecular architecture and play important roles in neuronal development. In particular, *LNX1* has been implicated in synapse maturation (GO:0060074), highlighting the involvement of the LNX family in key neurodevelopmental processes. While *LNX1* and *LNX2* display distinct spatiotemporal expression patterns, both are consistently expressed in the central nervous system, supporting a shared functional framework (Figure 8).

**Figure 9:**
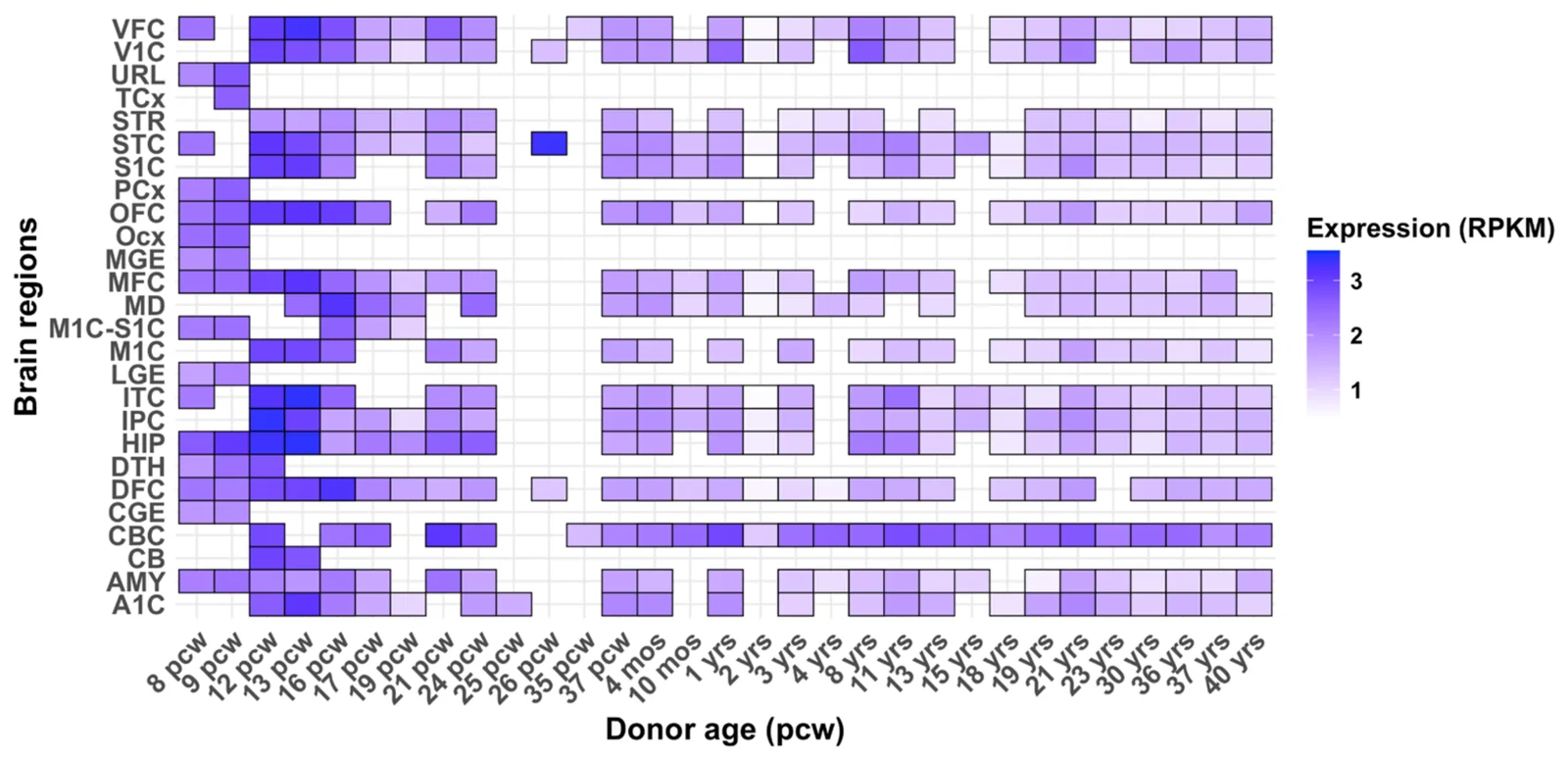
Developmental brain expression of LNX2. Data was retrieved from BrainSpan database and are measured in RPKM. The abbreviation used were: primary auditory cortex (core) (A1C); amygdaloid complex (AMY); cerebellum (CB); cerebellar cortex (CBC); caudal ganglionic eminence (CGE); dorsolateral prefrontal cortex (DFC); dorsal thalamus (DTH); hippocampus (hippocampal formation) (HIP); posteroventral (inferior) parietal cortex (IPC); inferolateral temporal cortex (area TEv, area 20) (ITC); lateral ganglionic eminence (LGE); primary motor cortex (area M1, area 4) (M1C); primary motor–sensory cortex (samples) (M1C-S1C); mediodorsal nucleus of thalamus (MD); anterior (rostral) cingulate (medial prefrontal) cortex (MFC); medial ganglionic eminence (MGE); occipital neocortex (Ocx); orbital frontal cortex (OFC); parietal neocortex (PCx); primary somatosensory cortex (area S1, areas 3, 1, 2) (S1C); posterior (caudal) superior temporal cortex (area 22c) (STC); striatum (STR); temporal neocortex (TCx); upper (rostral) rhombic lip (URL); primary visual cortex (striate cortex, area V1/17) (V1C); ventrolateral prefrontal cortex (VFC); post-conceptional week (pcw); months (mos); years (yrs). The complete developmental expression profile of LNX2 in the human brain is provided in Supplementary Table S4.

At the protein level, LNX1 and LNX2 show a sequence identity of approximately 53.2%, indicating a moderate degree of evolutionary conservation. Notably, the alanine residue at position 389 in LNX2 is conserved, suggesting functional importance. This residue is located within the PDZ2 domain (residues 339–422), a region known to mediate protein–protein interactions and substrate recognition. Given the critical role of PDZ domains in synaptic organization and signaling, alterations affecting conserved residues within this domain may have relevant functional consequences. LNX2 shows a marked developmental regulation, with peak expression during early prenatal stages (8–13 pcw), particularly in cortical regions and hippocampus (Figure 9).

Expression levels progressively decrease postnatally, stabilizing at lower levels in adulthood. This pattern suggests a primary role in early neurodevelopmental processes, such as neuronal differentiation and cortical organization. Consistently, LNX proteins have the ability to interact with and promote the degradation of the cell fate determinant protein NUMB [27]; on this basis, they are thought to play a role in modulating NUMB/NOTCH signalling during processes such as cortical neurogenesis. Together, these observations support a functional involvement during early brain development, with a more limited role in the mature brain. Furthermore, E3 ubiquitin ligases encoded by *LNX1* and *LNX2* are key regulators of protein turnover and signaling specificity, with emerging roles in immune function. Both proteins interact with the T-cell co-receptor CD8α via PDZ-mediated binding, promoting its ubiquitination, internalization, and degradation, thereby contributing to immune receptor homeostasis [28]. Notably, increasing evidence links immune dysregulation and neuroinflammation to NDDs [29]. In this context, the involvement of *LNX2* in ubiquitin-dependent regulation of immune receptors suggests a potential connection between immune and neurodevelopmental pathways. Although speculative, this observation provides additional biological plausibility for the role of *LNX2* in NDDs. Additional support for the potential neurodevelopmental relevance of LNX2 is provided by entries reported in the Human Gene Mutation Database (HGMD) (Table 2).

Although no definitive Mendelian phenotype has yet been assigned to LNX2, multiple rare coding variants have previously been reported in association with neurodevelopmental phenotypes, including ASD and developmental delay (DD). Notably, reported variants include both truncating and missense substitutions distributed across functionally relevant regions of the protein. While these observations do not establish causality, they independently support the emerging biological relevance of LNX2 in neuronal development and neurodevelopmental disease susceptibility. In this context, the identification of a rare homozygous missense variant affecting the PDZ2 domain in our patient provides additional support for the biological plausibility of LNX2 involvement in neurodevelopmental processes and supports the need for additional genetic and functional investigation.

Integrative transcriptomic and epigenomic analyses using datasets retrieved from Gene Expression Omnibus and PsychENCODE/PsychSCREEN provided additional support for the biological plausibility of *LNX2* involvement in neurodevelopmental pathways relevant to NDDs. Although bulk transcriptomic analyses across independent ASD postmortem brain datasets did not identify robust differential expression of LNX2, co-expression network analyses consistently positioned *LNX2* within biologically coherent neuronal transcriptional modules. In frontal cortex samples, *LNX2*-associated networks were enriched for genes involved in synaptic signaling, vesicle trafficking, intracellular transport, and cytoskeletal organization, including *PTPRT*, *KCND2*, *CADPS*, *SCAMP1*, *ATG5*, *WASL*, and *AP2A2* [32,33,34,35,36]. Correspondingly, Gene Ontology enrichment analyses demonstrated overrepresentation of pathways related to synaptic vesicle cycling, regulated exocytosis, axonal transport, intracellular trafficking, and microtubule-dependent transport, biological processes increasingly implicated in ASD- and ADHD-associated synaptic dysfunction. In particular, *AP2A2*, identified as a conserved co-expressed partner across independent datasets, further supported the potential involvement of LNX2 in neuronal trafficking pathways. *AP2A2*, a component of the adaptor protein-2 complex involved in clathrin-mediated endocytosis and synaptic vesicle recycling, emerged as a shared co-expressed gene across datasets [36,37]. The ASD datasets GSE28521 and GSE38322 used for this analysis were selected because they provided region-specific postmortem brain transcriptomic profiles with sufficient biological replication, enabling comparative analyses across distinct ASD-relevant brain regions. In contrast, exploratory analysis of the ADHD-related dataset GSE52889 revealed relatively stable peripheral LNX2 expression across untreated ADHD samples, unaffected controls, and methylphenidate-treated conditions. However, because GSE52889 consisted of pooled lymphoblastoid RNA samples lacking adequate biological replication, these findings were interpreted descriptively and do not exclude potential brain-specific functional effects of LNX2 in ADHD-related neurobiology.

Single-cell transcriptomic analyses derived from PsychENCODE/PsychSCREEN datasets additionally demonstrated preferential enrichment of LNX2 expression within deep-layer cortical excitatory projection neurons, particularly layer 5 intratelencephalic and near-projecting neuronal populations. These neuronal subtypes are critically involved in long-range corticocortical communication, executive circuitry, and higher-order cognitive processing, functions frequently disrupted in ASD and ADHD [38,39]. These findings provide additional evidence that LNX2 is embedded within neuronal transcriptional and regulatory networks relevant to neurodevelopmental biology.

The identified p.Ala389Thr variant affects the PDZ2 domain (residues 339–422), a functionally relevant region involved in mediating protein–protein interactions. Structural modelling and structural superposition indicated that the substitution did not induce major conformational changes, with the overall architecture of the PDZ2 domain remaining highly conserved. However, replacement of alanine with threonine introduced a polar hydroxyl-containing side chain predicted to establish an additional hydrogen bond with Thr417, resulting in a localized rearrangement of the hydrogen-bonding network. Because Ala389 is located outside the canonical PDZ ligand-binding groove, these observations are not expected to directly disrupt ligand recognition but may subtly influence the local conformational properties or dynamics of the PDZ2 domain. Notably, the hydrogen-bonding arrangement surrounding Thr417 was consistent with the experimentally resolved PDZ2 structure (PDB ID: 5E11), supporting the structural plausibility of the model used in this study. Nevertheless, these structural observations remain predictive and should be considered supportive rather than demonstrative of functional consequences, which will require experimental validation. These alterations, also supported by IUPred2 analysis, may be associated with changes in protein stability or interaction properties and could potentially impact the E3 ubiquitin ligase activity of LNX2.

Based on these considerations, we propose that *LNX2* represents a biologically plausible candidate gene whose role in neurodevelopment warrants further investigation. Importantly, the primary aim of the present study was not to establish a definitive disease–gene relationship, but rather to further explore the potential involvement of *LNX2* in neurodevelopment by integrating complementary lines of evidence.

Nevertheless, several limitations should be acknowledged. First, although the identified LNX2 variant was detected in the homozygous state in the proband and inherited from clinically unaffected non-consanguineous parents, phased haplotype reconstruction analysis was not available in the present study. Consequently, we were unable to investigate the presence of a potential shared ancestral haplotype, cryptic parental relatedness, or identity-by-descent mechanisms that may underline the occurrence of this rare homozygous variant. Future genome-wide haplotype and segregation analyses may help clarify the genetic background surrounding the identified variant. Second, NDDs are characterized by substantial genetic heterogeneity and multifactorial etiologies involving both genetic and environmental contributors. Therefore, we cannot exclude the involvement of additional genetic, epigenetic, or non-genetic factors not captured through WES analysis. Accordingly, the identified homozygous LNX2 variant should not be interpreted as a definitive monogenic cause of the disorder, but rather as a potential contributory factor within a broader neurodevelopmental susceptibility framework. Moreover, no functional studies, such as protein–protein interaction assays, ubiquitination assays, signaling analyses, or expression studies, were performed to validate the predicted effects of the p.Ala389Thr variant. Additional independent cases and genotype–phenotype correlations will be necessary to clarify the contribution of LNX2 to human disease.

## Figures and Tables

**Table 1:** In silico prediction for the c.1165G>A p.Ala389Thr variant in LNX2 gene. Numerical scores correspond to the original output of each prediction algorithm, whereas the “Prediction” column reports the classification provided by VarSome. The principal score thresholds for the individual algorithms are as follows: CADD, PHRED score ≥ 20; SIFT/SIFT4G, <0.05; PolyPhen-2, >0.909 (probably damaging); FATHMM-XF, >0.5; MutPred2, ≥0.5; DANN, scores closer to 1 indicate higher pathogenicity; EIGEN/EIGEN-PC, higher positive values indicate greater predicted deleteriousness; LIST-S2, PrimateAI, VARITY_R and VARITY_R_LOO, higher scores indicate increased likelihood of pathogenicity; Mutation Assessor: low (<1.9), medium (1.9–3.5), and high (>3.5) functional impact; BLOSUM100: positive scores indicate evolutionarily tolerated substitutions, whereas negative scores indicate less tolerated amino acid substitutions.

Algorithm	Prediction	Score
CADD	Pathogenic Supporting	27.7999
SIFT	Uncertain	0.008
SIFT4G	Uncertain	0.01
PrimateAI	Uncertain	0.7357
LIST-S2	Uncertain	0.9599
Mutation Assessor	Uncertain	2.745 (×8)
FATHMM-XF	Uncertain	0.7463
DANN	Uncertain	0.9985
EIGEN	Uncertain	0.4459
PolyPhen-2	Probably damaging	0.970
EIGEN PC	Uncertain	0.4182
BLOSUM100	Uncertain	−1
MutPred2	Uncertain	0.6565
VARITY_R	Uncertain	0.5298 (×8)
VARITY_R_LOO	Uncertain	0.5298 (×8)

**Table 2:** Reported *LNX2* variants associated with neurodevelopmental phenotypes.

Accession	Variant	Protein Change	Class	Phenotype	Reference
CM2230693	c.1396G>T	p.Glu466Ter	DM?	ASD	[30]
CM2051548	c.1797C>T	p.His599His	DM?	DD	[31]
CM2072851	c.1977C>T	p.Thr659Thr	DM?	DD	[31]

Note: Variants were retrieved from HGMD Professional 2026.1 and include previously reported LNX2 alterations associated with autism spectrum disorder (ASD) and developmental disorders (DDs). Abbreviations: ASD, autism spectrum disorder; DD, developmental disorder; DM?, likely disease-causing mutation according to HGMD classification.

## Data Availability

The data presented in this study are available upon request from the corresponding author.

## Supplementary Materials

The following supporting information can be downloaded at: https://www.mdpi.com/article/10.3390/genes17080849/s1, Figure S1. Structural and sequence comparison of wild-type and p.Ala389Thr LNX2; Figure S2. Regional LNX2 expression across independent ASD transcriptomic datasets; Table S1. Complete list of LNX2-specific co-expressed genes identified through co-expression analysis of the GSE28521 dataset; Table S2. Complete list of LNX2-specific co-expressed genes identified through co-expression analysis of the GSE38322 dataset; Table S3. Complete VarSome output of the in silico pathogenicity prediction analyses for the identified LNX2 variant; Table S4. Developmental brain expression profile of LNX2. Complete expression data for LNX2 across human brain developmental stages used in the present study; File S1: LNX2_wild_type_Best_mode; File S2: LNX2_Ala389Thr_Best_model; File S3: LNX2_PDB_5e22.

## Abbreviations

The following abbreviations are used in this manuscript:

ACMG	American College of Medical Genetics
ADHD	Attention-deficit/hyperactivity disorder
ASD	Directory of open access journals
IGV	Integrative Genomics Viewer
NDD	Neurodevelopmental disorder
NICD	NOTCH intracellular domain
ODD	Oppositional defiant disorder
RMSD	Root mean square deviation
UPS	Ubiquitin–proteasome system
VUS	Variant of uncertain significance
WES	Whole exome sequencing
