# Supplementary material for: Deciphering the Role of LNX2 as a Potential Contributor to Neurodevelopmental Disorders
Source: Genes (Basel). 2026 Jul 23;17(8):849. doi: 10.3390/genes17080849 (PMC13511767; doi:10.3390/genes17080849)
Supplement: Supplementary file 1 [file genes-17-00849-s001.zip › Figure S1 and S2.pdf]

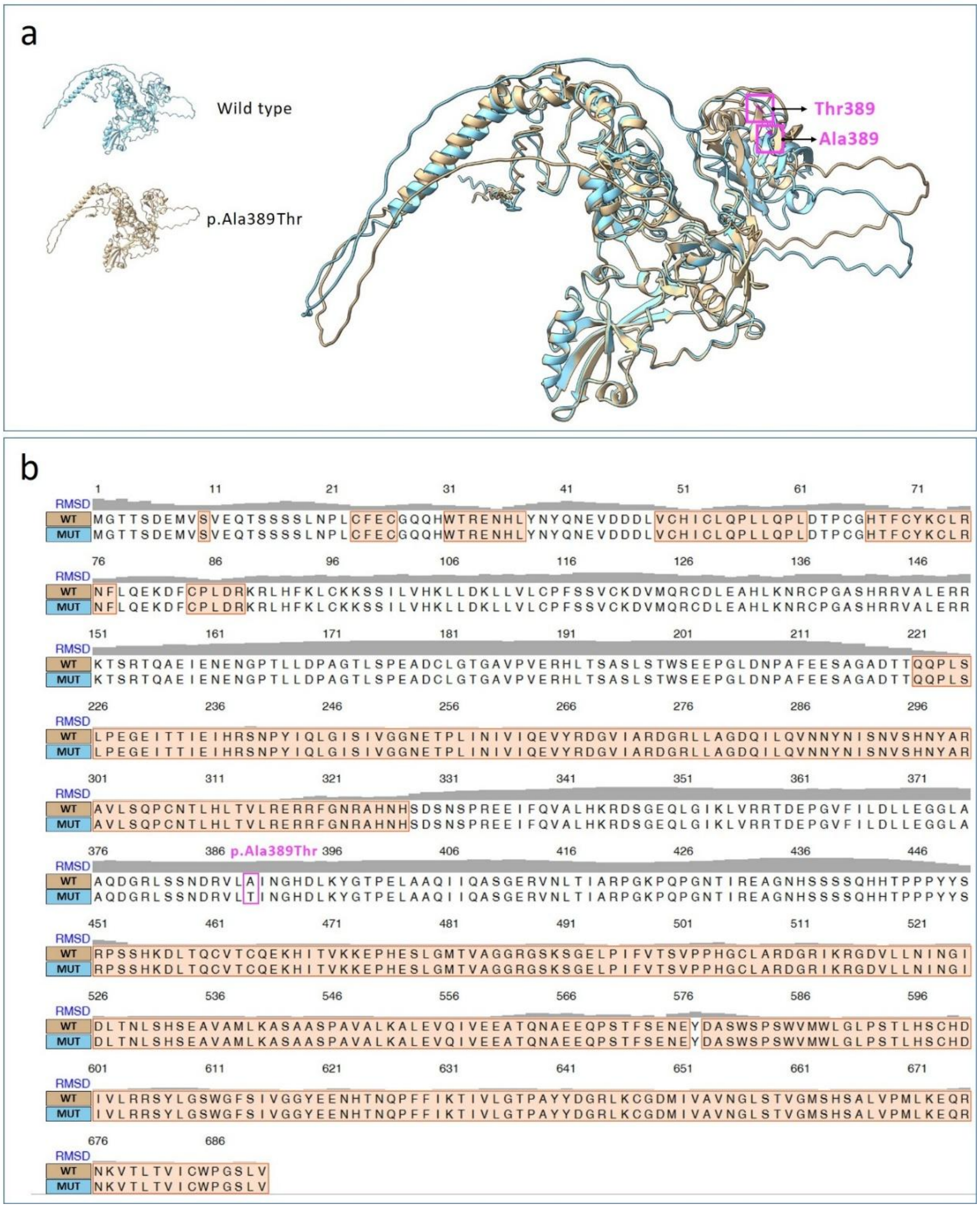

Figure S1. Structural and sequence comparison of wild-type and p.Ala389Thr LNX2. (a) Structural superposition of the AlphaFold3-predicted wild-type (cyan) and p.Ala389Thr mutant (beige) LNX2 proteins. The mutation site is highlighted by the magenta squares, showing the substitution of Ala389 with Thr389 within the PDZ2 domain. (b) Sequence alignment of wild-type and mutant LNX2 proteins annotated with per-residue structural deviation (RMSD). Conserved regions are highlighted, while the mutation site at position 389 is indicated.

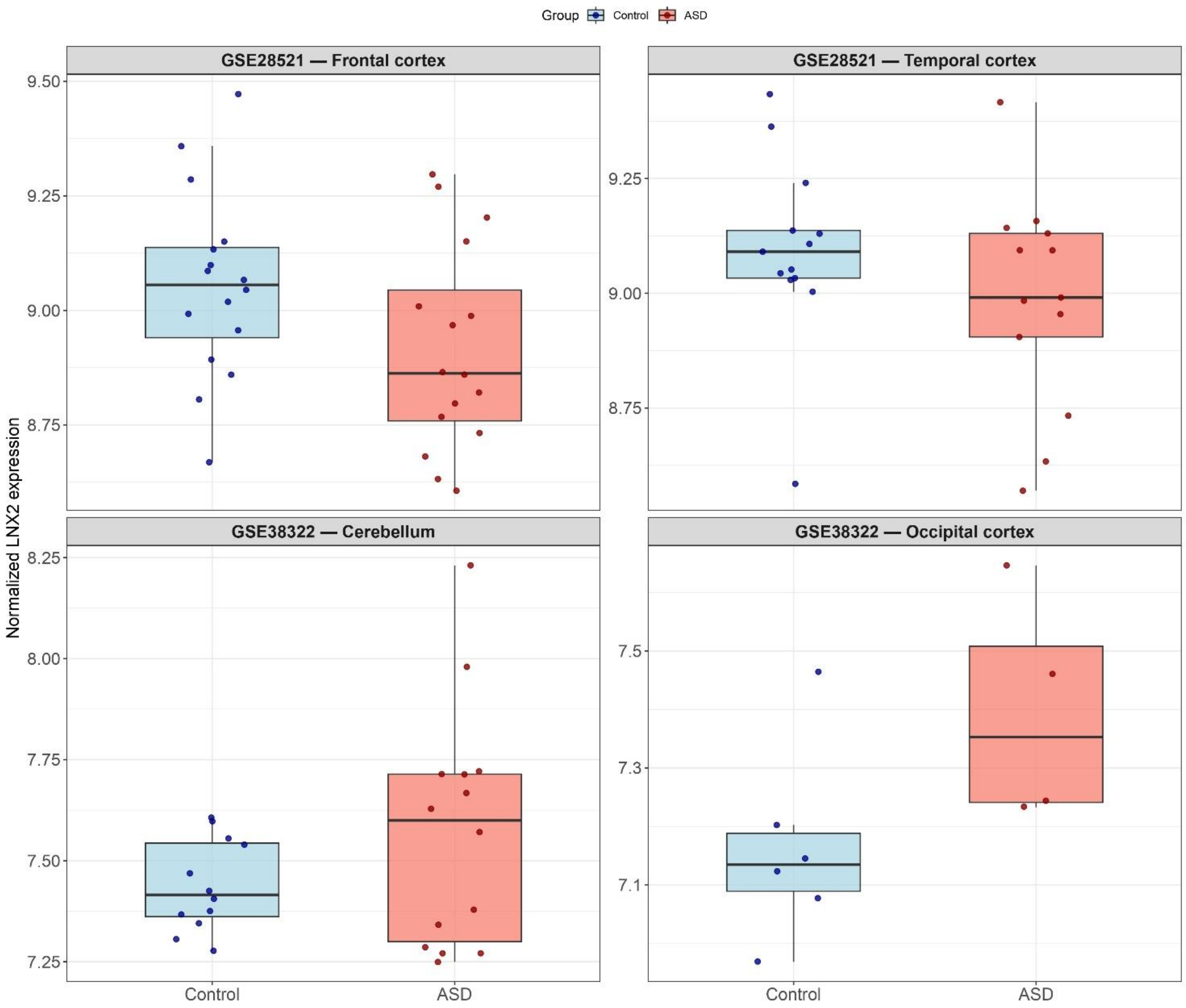

Figure S2. Regional LNX2 expression across independent ASD transcriptomic datasets. Normalized LNX2 expression levels are shown in ASD and control postmortem brain samples from GSE28521 (frontal and temporal cortex) and GSE38322 (cerebellum and occipital cortex). LNX2 displayed modest region-dependent expression trends across ASD brain regions, with reduced cortical expression in GSE28521 and increased cerebellar/occipital expression in GSE38322, although these effects did not reach statistical significance.
